# Deletion of glucose oxidase changes the pattern of organic acid production in *Aspergillus carbonarius*

**DOI:** 10.1186/s13568-014-0054-7

**Published:** 2014-08-15

**Authors:** Lei Yang, Mette Lübeck, Peter S Lübeck

**Affiliations:** 1 Section for Sustainable Biotechnology, Aalborg University Copenhagen, A. C. Meyers Vænge 15, Copenhagen, DK-2450, SV, Denmark

**Keywords:** Aspergillus carbonarius, Citric acid, Glucose oxidase, Gluconic acid, Malic acid

## Abstract

*Aspergillus carbonarius* has potential as a cell factory for the production of different organic acids. At pH 5.5, *A.carbonarius* accumulates high amounts of gluconic acid when it grows on glucose based medium whereas at low pH, it produces citric acid. The conversion of glucose to gluconic acid is carried out by secretion of the enzyme, glucose oxidase. In this work, the gene encoding glucose oxidase was identified and deleted from *A. carbonarius* with the aim of changing the carbon flux towards other organic acids. The effect of genetic engineering was examined by testing glucose oxidase deficient (*Δgox*) mutants for the production of different organic acids in a defined production medium. The results obtained showed that the gluconic acid accumulation was completely inhibited and increased amounts of citric acid, oxalic acid and malic acid were observed in the *Δgox* mutants.

## Introduction

With depletion of crude oil and increased environmental concern, biologically based processes for producing organic acids that can be used as building blocks in the chemical industries have begun to raise attention in recent years (Holladay et al. [2007]). It is widely accepted that a suitable industrial strain for organic acid production is one of the key factors affecting the feasibility of the entire production process. Filamentous fungi have been well studied as cell factories for different production of organic acids for decades because they exhibit excellent abilities to utilize a variety of carbon sources and naturally accumulate high amount of specific organic acids under stressed conditions, like citric acid production by *Aspergillus niger* and malic acid production by *Aspergillus flavus* (Battat et al. [1991]; Papagianni et al. [1999]). Although the mechanisms of organic acid accumulation by different fungi have not been fully understood, many successful attempts have been made to improve organic acid production in filamentous fungi by using genetic modification and production optimization. In this study, *Aspergillus carbonarius*, which has a close phylogenetic relationship to *Aspergillus niger* (Thom and Currie [1916]) was selected to exploit its potential as a new cell factory for production of different organic acids. It resembles *A. niger* in many features, including morphology and high capacity of producing citric acid (Ghareib [1987]; Joosten et al. [2001]; Weyda et al. [2014]), which creates the possibility to apply the knowledge accumulated for *A. niger* directly to metabolic engineering of *A. carbonarius* for production of organic acids.

In organic acid production using filamentous fungi, pH is an important parameter. It has been reported that low pH is necessary for obtaining high yields of citric acid using *A. niger*, and reversely pH around 5 ~ 6 is preferred for producing malic acid using *A. flavus* and fumaric acid using *Rhizopus oryzae* (Peleg et al. [1988a]; Xu et al. [2012]). The production pattern of organic acids can also be changed dramatically in filamentous fungi by adjusting pH during cultivation. In *A. niger*, production of citric acid was dramatically suppressed at near neutral pH as gluconic acid started accumulating in high amount (Bercovitz et al. [1990]; Goldberg et al. [2006]). In this study we investigated the effect of deleting the glucose oxidase in *Aspergillus carbonarius* for the purpose of organic acid production at pH 5–6. At this pH range, the fungus accumulates high amounts of gluconic acid, presumably due to secretion of glucose oxidase, whereby the fungus quickly converts glucose into gluconic acid outside the cell thus preventing further metabolism of glucose (Mischak et al. [1985]). The aim of this study was therefore to eliminate the gluconic acid production in order to increase the carbon flux towards other organic acids. The hypothesis was: by eliminating gluconic acid production, *A. carbonarius* would increase 1,4-dicarboxylic acid production. Deletion of the gene encoding the glucose oxidase (*gox*) was conducted in *A. carbonarius* to suppress the conversion of glucose to gluconic acid. However, low frequency of homologous recombination in filamentous fungi often leads to very limited gene targeting efficiency in fungal transformation. Therefore, a Ku complex deficient strain of *A. carbonarius* (Gallo et al. [2012]), which is supposed to dramatically increase the homologous recombination frequency due to the inactivation of Ku complex, was selected for this work.

## Materials and methods

### Strains and culture medium

A Ku deficient strain, KB1039 (*Δkus*A, obtained from K. Bruno, PNNL, US), which is also uracil auxotrophic (*ΔpyrG)*, of the wild type *A. carbonarius* 5010 (ATCC® MYA-4641™) (Gallo et al. [2012]), was used as the parental strain to construct the *Δgox* mutants. Both the *A. carbonarius* KB1039 (*Δkus*A) and the wild type 5010 was cultured at PDA (potato dextrose agar) medium at 30°C and for the Ku deficient strain, the medium was supplemented with uracil and uridine, each at the final concentration of 2 mM.

### Identification of the glucose oxidase gene in *A. carbonarius*

Due to lack of information about glucose oxidase (GOX) genes in *A. carbonarius*, the sequence of the *gox* gene in *A. niger* (accession no. X16061.1) was selected to identify the orthologous *gox* gene in *A. carbonarius* based on the close phylogenetic relationship between *A. niger* and *A. carbonarius.* One sequence with high identity was identified in the alignment hit as a putative *gox* gene in *A. carbonarius.* The sequence containing extension of 1000 bp from both 5’ and 3’ flanking regions of the *gox* encoding gene sequence was also identified with the purpose of using these 2 kb sequence flanking regions surrounding the putative *gox* gene for deletion of the gene. The sequence was submitted to GenBank with the accession no. KF741791.

### Plasmid construction for gene knock-out

The genomic DNA was isolated from wild type *A. carbonarius* 5010 by using phenol-chloroform extraction (Andreou [2013]) and used as template to amplify 5’ and 3’ flanking regions of the *gox* gene with primers containing uracil overhang (Table 1). Since the gene knock-out was carried out in a Ku deficient strain, it was not necessary to have very long fragments to increase the frequency of homologous recombination. It is reported that fragments about 1 kb both upstream and downstream to a target gene could provide efficient homologous recombination in *A. niger* (Meyer et al. [2007]). Therefore, the size of fragments amplified from *A. carbonarius* was between 900 and 1000 bp. The PCR reaction was set up in 50 μL reaction volume: 5 μL 10X *pfu* turbox buffer; 1 μL 10μM dNTP; 2.5 μL 10 uM forward and reverse primer; 1 μL pfu turbo cx polymerase (Agilent); appropriate amount of DNA template and water added up to 50 μL. The PCR program was as follows: Initial denaturing step at 95°C for 3 min; 25–30 cycles of denaturing step at 94°C for 30s; annealing step at 55-65°C for 30s; elongation step at 72°C for specific amount of time calculated by the size of desired fragments (1 min/kb); final elongation step at 72°C for 5 minutes. The *pfu* turbocx polymerase was only used to amplify the DNA fragment with primers with uracil overhang in simpleUSER cloning (Hansen et al. [2014]), whereas all other PCR reactions were carried out using RUN (taq) polymerase (A&A biotechnology). The plasmid pSB414 was designed and constructed for simpleUSER cloning (Hansen et al. [2014]) and contains the following genetic elements: *gpdA* promoter, *trpC* terminator and *pyrG* gene, including a specific cassette facilitating simpleUSER cloning. The cassette was activated by the restriction enzyme *Pac*I and the nicking enzyme *Nb.BbvCI* to generate the complementary overhang to the target fragments. The target fragments were cloned into the plasmid through self-assembly followed by transformation of *E. coli* with the plasmid for further propagation using standard procedures.

**Table 1:** Primers used in this research

**Name**	**Sequence (5’ → 3’)**	**Annotation**
**Gox upU Fw**	*GGGTTTAAU*TCTCCTTGTGCTGACCAACCG	USER cloning of gene *gox* upstream
**Gox upU Rv**	*GGACTTAAU*GTTTACCAATCCCGCCGCGTC	USER cloning of gene *gox* upstream
**Gox downU Fw**	*GGCATTAAU*AGGTGAGATGGAGTTGTTG	USER cloning of gene *gox* downstream
**Gox downU Rv**	*GGTCTTAAU*TTGGGATGGGTAGGGTATT	USER cloning of gene *gox* downstream
**Gox Fw1**	GCCCTGCCACACTACATCCG	Amplify internal sequence of gene *gox*
**Gox Rv1**	TCGCCACAGCCGAGATCCTT	Amplify internal sequence of gene *gox*
**Gox Fw2**	GCTGCCAATCCTTCGGTCCA	Amplify internal sequence of gene *gox*
**Gox Rv2**	TAGTCGCCAAAGGTCTCGTT	Amplify internal sequence of gene *gox*
**Gox Fw3**	AACAACCTCACCCACCAGAG	Amplify the sequence containing gene *gox*
**Gox Rv3**	ACCATTGAAGTGGCAGGAAC	Amplify the sequence containing gene *gox*

### Protoplast transformation

Protoplasts of *A. carbonarius* were made from young mycelium harvested after overnight growth in YPD medium. The cell walls were degraded by 60 mg/ml of the commercial product Vino Taste Pro (Novozymes A/S) in protoplasting buffer (1.2 M MgSO_4_, 50 mM Phosphate Buffer, pH 5.0) for approx. 4 hours. Protoplasts were filtered and purified from the mixture, suspended in STC buffer (1.0 M sorbitol, 50 mM Tris, 50 mM CaCl_2_ pH 8.0) and counted with appropriate dilution folds in a haemacytometer. The final concentration of protoplasts for aliquots was adjusted to 2×10^7^/mL. Protoplast transformation was carried out by adding 5 μg plasmids in 100 μl protoplast suspension and incubated on ice for 15 minutes followed by incubation for 15 minutes at room temperature after adding 1 mL of 40% PEG. The mixture was transferred into 10 mL minimum medium (Gallo et al. [2012]) with 1 M sorbitol at 30°C for 1 hour with agitation at 80 rpm in the incubator shaker (KS 4000 I control, IKA). Then the cells were concentrated by centrifugation for 5 minutes at 800 × g, re-suspended in minimal medium containing 1 M sorbitol and 0.8% agar and poured into petri-dishes. Next day, a second layer of the same medium was poured on the top. The plates with potential transformants were incubated at 30°C for at least 3 days until transformants appeared.

Sporulating transformants were inoculated by streaking out on PDA medium and incubated at 30°C overnight. Single colonies were identified and picked out to verify the deletion of the target gene by extracting the genomic DNA from the transformants and amplifying the fragments with expected size in PCR. The transformants were further transferred to normal PDA plates and preserved for further steps.

### Growth conditions

Spores of fungal transformants were harvested from PDA plates after 5–7 days of cultivation at 30°C, and collected through sterilized Miracloth (EMD Millipore, USA) in sterile 0.05 M phosphate buffer pH 6.8 in a 15 mL falcon tube. The spores were counted in a haemacytometer and then inoculated into 50 mL falcon tubes containing 10 mL pre-culture medium (3.6 g/L yeast extract and 10 g/L peptone). The final concentration of spores in the pre-culture medium was approximately 1×10^5^/mL. Pre-cultivation was carried out at 30°C with agitation of 250 rpm for 2 days. Pellets formed in the pre-culture medium were then transferred into production medium by filtering the pre-culture medium through Miracloth, and all the pellets on the top were collected and transferred into the production medium, which was modified from the production medium C described by Peleg et al. [1988a], [b]: Glucose, 100 (g/L); (NH_4_)_2_SO_4_, 2 (g/L); KH_2_PO_4_, 0.15 (g/L); K_2_HPO_4_, 0.15 (g/L); MgSO_4_ 7H_2_O, 0.1 (g/L); CaCl_2_ 2H_2_O, 0.1 (g/L); NaCl, 0.005 (g/L); FeSO_4_ 7H_2_O, 0.005 (g/L), 0.1 g/L ZnSO_4 _and CaCO_3_, 60 (g/L) (Peleg et al. [1988a]). Cultivation was carried out in 100 mL flasks containing 20 mL production medium at 30°C with agitation of 180 rpm. The cultivation time varied from 7 to 10 days. The pre-culture and acid production was carried out in triplicates and pH was kept at 5.5 for the entire procedure.

### Analysis of extracellular metabolites

Samples taken from organic acid fermentation were acidified with 72% sulfuric acid to a final concentration of 5% in order to precipitate the calcium ion in form of calcium sulfate and exchange the organic acids back to liquid phase. The acidified samples were incubated at 80°C for at least 15 minutes to complete the reaction. After incubation, pH of the samples should be lower than 2 and was checked by pH indicator paper. The acidified samples were then centrifuged at 10,000 rpm for 1 minute and the supernatant was used for HPLC analysis. The analysis for sugar and organic acids were carried out in Aminex 87H column (Biorad) at 60°C by using HPLC mobile phase at a flow rate of 0.6 mL/minute. The HPLC samples were kept at 4°C in the machine during the analysis process and then stored at −20°C. The measurements of L-malic acid and D- gluconic acid in the samples were carried out respectively with L-malate (L-malic acid) kit and D-gluconate kit as described by the manufacturer (Megazyme).

## Results

### Protoplast transformation and deletion of the *gox* gene from *A. carbonarius*

**Figure 1:**
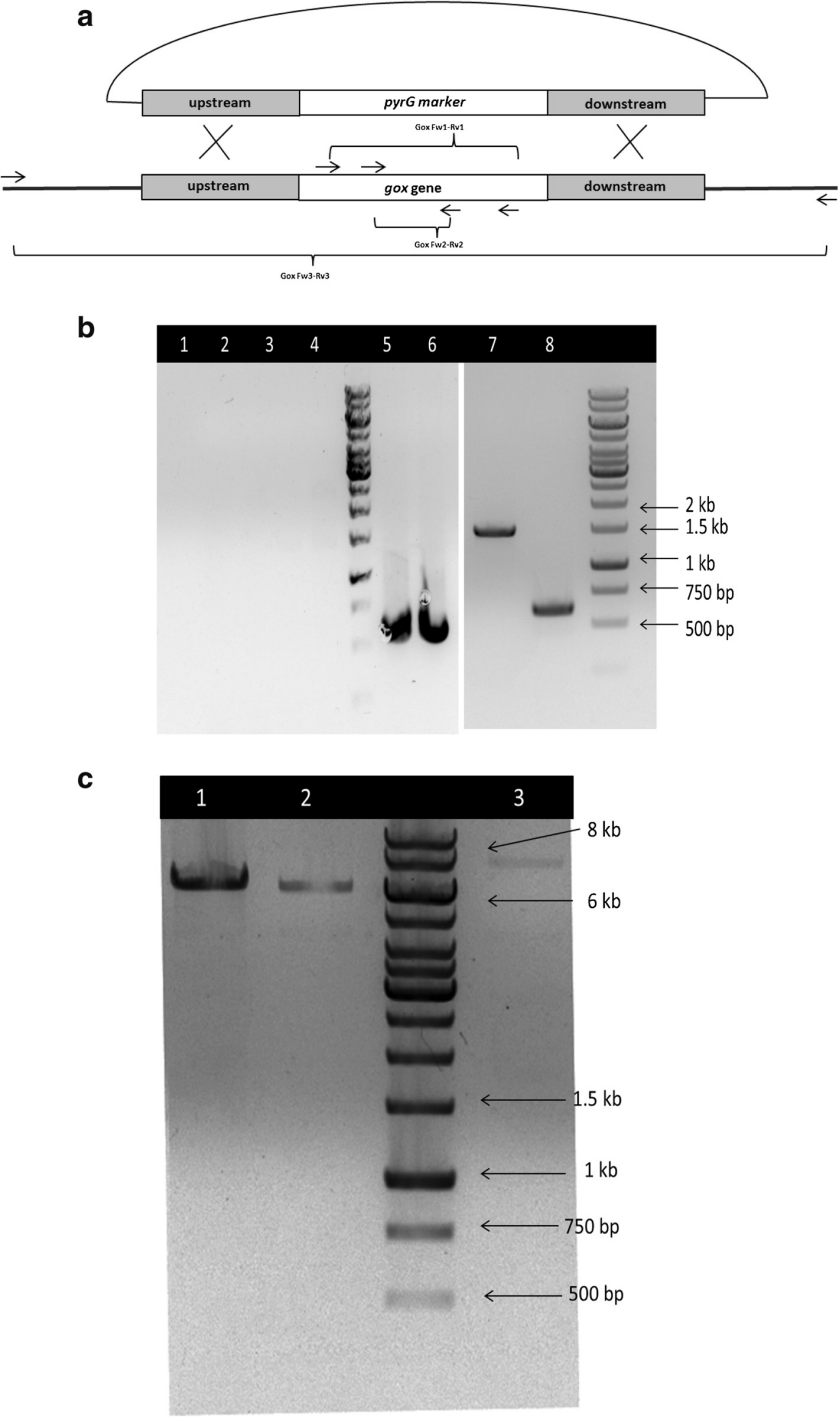
**Verification of deletion of the*gox*gene in transformant 4 and 5 (a) Disruption of*gox*gene and primer binding sites (b) Amplification of internal sequence with primer Gox Fw1-Rv1 and Fw2-Rv2.** Lane 1–2, *Δgox* transformant 4; lane 3–4, *Δgox* transformant 5; lane 5–6, ITS sequence amplified from *Δgox* transformant 4 and 5; lane 7–8, internal fragments of the gox gene from the wildtype strain (~1,5 kb and 0,6 kb) **(c)** Amplification of the *gox* gene containing region with the external primers Gox Fw3 and Rv3. Lane 1–2, amplified fragments from gox transformants 4 and 5. (~6.2 kb). Lane 3, amplified fragment from wild type (~7.7 kb).

Protoplast transformation was carried out with circular pSB414*gox* plasmids. Two *Δgox* transformants were obtained and checked for the deletion of the putative *gox* gene by 3 different pairs of primers (Table 1). Two pairs of primers (Gox Fw1-Rv1 and Fw2-Rv2) amplified the internal sequence of the putative *gox* gene, and the third pair of primers (Gox Fw3-Rv3) was designed to amplify the putative *gox* gene together with the flanking regions in order to check the replacement of the *gox* gene (Figure 1a). As shown in Figure 1b, no fragments could be amplified by the two pairs of internal primers in PCR, indicating that the *gox* gene was deleted. In PCR with external primers, a fragment at approx. 6.2 kb was amplified from both of the transformants, which indicated that the putative *gox* gene had been successfully replaced by the marker gene, since the length of the original sequence containing the *gox* gene was approx.7.7 kb (Figure 1c). A PCR amplifying the ITS region (Bulat et al. [2000]) confirmed the quality of the genomic DNA (Figure 1b). The size of all the amplified DNA fragments were estimated by comparing with 1 kb DNA ladder (Figure 1b).

### Effect of deletion of the *gox* gene on gluconic acid production by *A. carbonarius*

**Figure 2:**
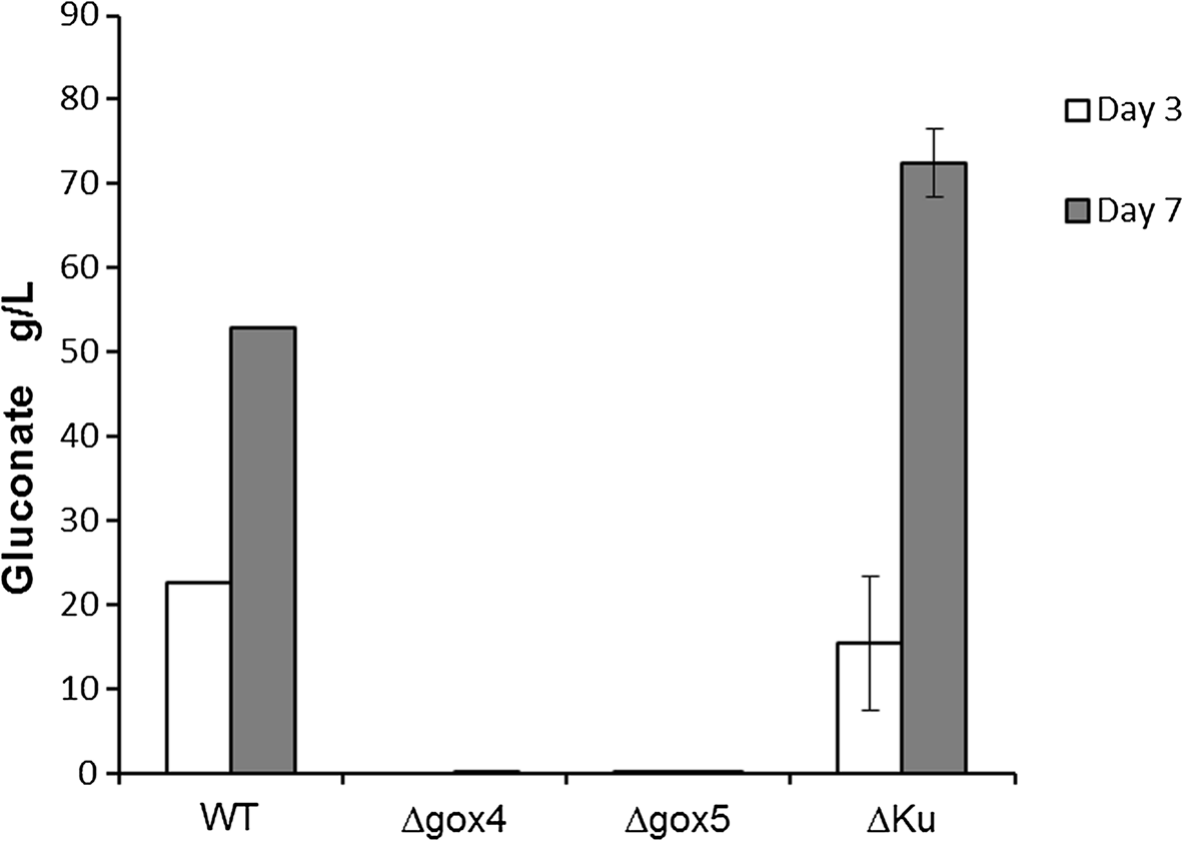
**The results of fermentation test on*Δgox*mutants in malic acid production medium.** Only one sample from wild type strain was measured for gluconic acid production, whereas all others were tested in triplicates.

**Figure 3:**
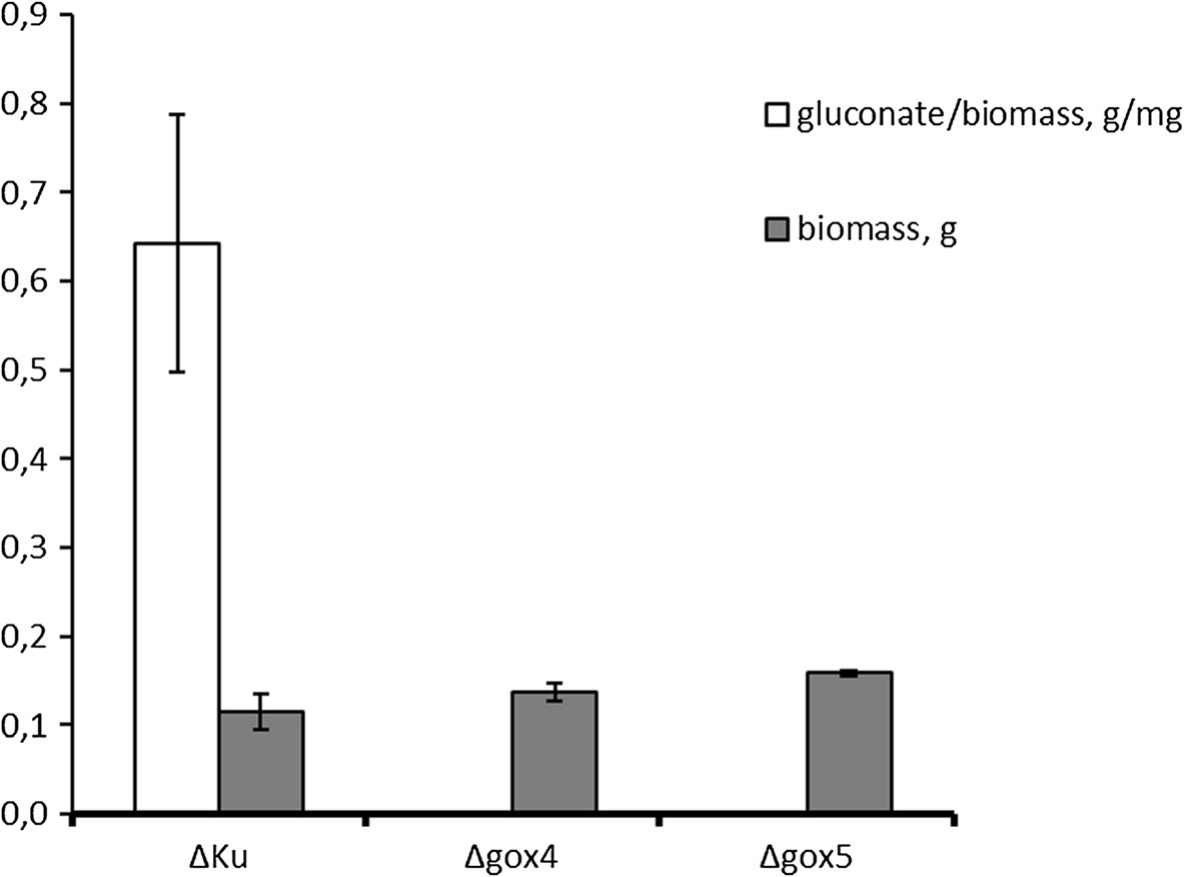
**The yield of gluconic acid production based on the biomass growth.** The yield was calculated based on the results from the samples taken on day 7.

The *gox* gene deleted from *A. carbonarius* is supposed to play a key role in the formation of gluconic acid. In order to evaluate the effect of this genetic modification on gluconic acid production, growth experiments were carried out with the *Δgox* mutants for 7 days at pH 5.5 by employing the *A. carbonarius* 5010 and KB1039 (*Δkus*A) as control. As shown in Figure 2, the KB1039 (*Δkus*A) and wild type strain (5010) produced high amount of gluconic acid (72 g/L and 53 g/L respectively) in the production medium after 7 days, and both of the *Δgox* transformants only produced 0.1 g/L gluconic acid by the end of the growth experiment. However, in order to confirm that the low gluconic acid production was not caused by different growth rates of the *Δgox* mutants, the biomass of the *Δgox* mutants was determined after the growth experiment. As shown in Figure 3, the *Δgox* mutants produced similar amount of biomass as the parent strain, which indicated that they grew equally well under the same conditions. The result confirmed that deletion of the *gox* gene can effectively inhibit the formation of gluconic acid by *A. carbonarius*.

### Analysis of extracellular metabolites of the *Δgox* mutants in the acid production media

**Figure 4:**
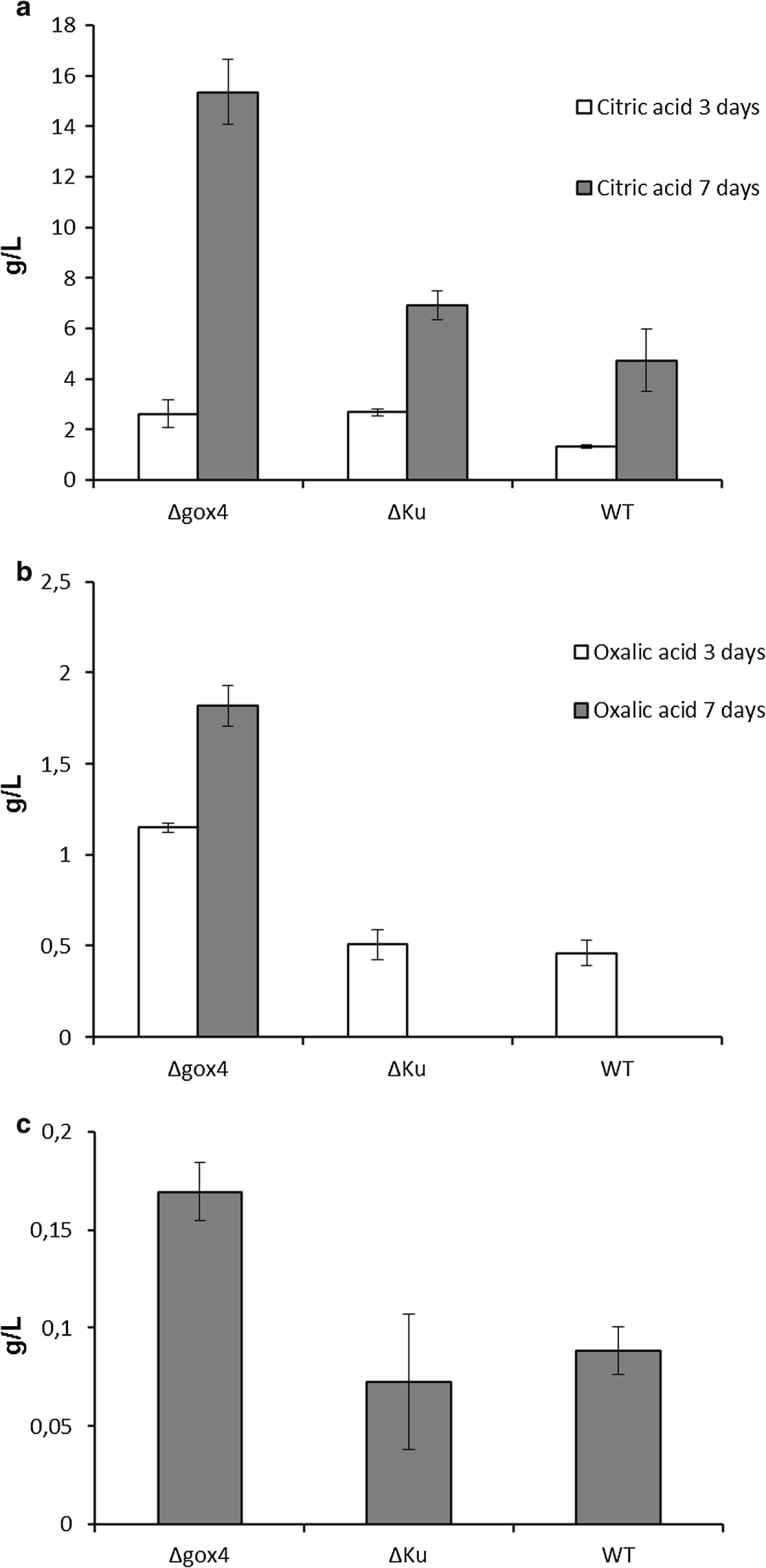
**The effect of gene deletion on the production of organic acids by*A. carbonarius.*(a)** The concentration of citric acid in the fermentation medium after day 3 and 7 **(b)** The concentration of oxalic acid in the fermentation medium after day 3 and 7 **(c)** The concentration of malic acid in the fermentation medium after day 7.

Cultivation with the selected *Δgox* mutants was carried out in shaking flasks at pH 5.5 to investigate the effect of the *gox* gene deletion on production of organic acids in *A. carbonarius.* However, due to the accumulation and consumption of gluconic acid by wild type *A. carbonarius*, it is difficult to use glucose as the sole carbon source to compare the performance of wild-type strain with the *Δgox* mutant during growth. The evaluation of organic acid production was carried out based on the concentration of extracellular acid products in the cultivation broth. As shown in Figure 4a, the concentration of citric acid was dramatically increased after 7 days cultivation in the *Δgox* mutants compared with the wild type 5010 and the parent KB1039 (*Δkus*A) strains. In addition, an accumulation of oxalic acid was also observed in the *Δgox* mutants during the cultivation (Figure 4b) and the production of malic acid also increased 2.4 and 1.8 folds, respectively, compared with the parent strain and the wild type strain (Figure 4c).

## Discussion

In the present work, a glucose oxidase gene (*gox*) in *A. carbonarius* involved in gluconic acid production was identified and deleted*.* In order to achieve a high gene targeting efficiency to facilitate the process of deleting the *gox* gene, a Ku deficient strain KB1039 (*Δkus*A) was selected to construct *Δgox* transformants. The Ku complex including Ku70 and Ku80 has been reported to play an essential role in the non-homologous end joining pathway (Dudášová et al. [2004]). Deletion of the Ku encoding gene can inactivate the non-homologous recombination mechanism and increase the frequency of homologous recombination (Kooistra et al. [2004]; Meyer et al. [2007]; Ninomiya et al. [2004]). In this study it was shown that the transformed DNA solely was integrated via homologous recombination in all the obtained transformants which proved that Ku deficient strain could be used as an efficient tool in gene targeting and study of gene function in *A. carbonarius.* By deleting the *gox* gene in a *Ku* deficient strain, it was also shown that the production of gluconic acid was dramatically reduced in *Δgox* mutants. Evaluation of growth experiments after 7 days of cultivation showed that the mutants produced increased amounts of malic acid and citric acid as well as an accumulation of oxalic acid (Figure 4).

Since the aim of this work was to investigate the effect of deleting glucose oxidase in *A.carbonarius* on the production of organic acids at a pH where the glucose oxidase is expressed, the cultivation was carried out at pH 5.5. Under this condition, *A. carbonarius* was capable of producing high amount of gluconic acid in media containing high concentration of glucose, resulting in a fast depletion of glucose, and it was shown that the elimination of glucose oxidase had an impact on production of other organic acids. Sugar concentration is considered as an important factor in organic acid production for filamentous fungi and has been used e.g. for citric acid production by *A. niger,* malic acid by *A. flavus,* and fumaric acid and lactic acid by *R. oryzae* (Alvarez-Vasquez et al. [2000]; Peleg et al. [1988b]; Ding et al. [2011]). The effect of high sugar concentration on organic acid production has been investigated in *A. niger* for citric acid production. These studies indicate that high sugar concentration seems to repress the α-keto-glutarate dehydrogenase in *A. niger* and increases the intracellular concentration of fructose-2,6-bisphosphate which leads to high yield of citric acid production by activating phosphofructokinase (PFK1) (Kubicek-Pranz et al. [1990]; Papagianni [2007]). Reversely, the conversion of glucose to gluconic acid will decrease the sugar concentration very fast during cultivation and further eliminate the effect of high sugar concentration. This assumption was supported in this study by comparing the pattern of organic acid production in the Δ*gox* mutants with the parental strain and the wild type strain after 3 days of cultivation. In the early phase of acid production, the glucose concentration still remained high although part of the glucose was already converted to gluconic acid. Therefore, the pattern of organic acids was similar among the *Δgox* mutants and the parental and wild type strains. However, when the glucose concentration in the media became much lower for the parental and wild type strains than for the *Δgox* mutants in the later phase of cultivation due to the accumulation of gluconic acid, the pattern of production of organic acid in the parental and wild type strains started varying from the *Δgox* mutants. The *Δgox* mutants produced higher amount of malic acid and citric acid compared with the parental and wild-type strains, and after 7 days of cultivation, production of oxalic acid was also observed in the *Δgox* mutants.

The enhanced production of citric acid and malic acid as well as accumulation of oxalic acid in the *Δgox* mutants must be a result of a higher carbon flux through the Glycolysis. Citric acid is solely produced through the TCA cycle*,* and the enhanced production indicates an improvement of the carbon flux into relevant pathways in *A. carbonarius* including glycolysis and TCA cycle. Moreover, the accumulation of oxalic acid might imply an elevated carbon flux towards cytosolic oxaloacetate reduction pathway which converted oxaloacetate to malate in the cytosol. Although, the oxalic acid producing pathway has not been well studied in *A. carbonarius*, it has been shown in *A. niger*, that oxalic acid is mainly produced by hydrolyzing oxaloacetate into oxalate and acetate by the enzyme oxaloacetase located in the cytosol (Ruijter et al. [1999]). Due to the close phylogenetic relationship between *A. niger* and *A. carbonarius*, it could be concluded that the accumulation of oxalic acid was most probably attributed to an increase of oxaloacetate in the cytosol, which was also in accordance with the increased production of malic acid during cultivation. Therefore, the high sugar concentration and improved carbon flux towards acid producing pathway might result in the different organic acid pattern in *Δ*gox mutant.

It is very likely that malic acid in *A. carbonarius*is transiently produced but not capable of accumulating at high concentration, especially in the *Δgox* mutant, the enhanced carbon flux towards the cytosolic oxaloacetate reduction pathway was supposed to result in a much higher concentration of malic acid during cultivation, but this was not achieved in our experiments. Malate, as an important intermediate of the TCA cycle, plays multiple roles in cell metabolism. A dramatically increase of intracellular malate concentration might influence the cell function and therefore result in immediately reduction via other pathways. The transiently produced malate may not be exported and accumulated during cultivation since *A. carbonarius* is able to produce high amount of citric acid and the malate instead may be directly used in intracellular anti-port of citrate and malate across the membrane of mitochondria as it is suggested for *A. niger* (de Jongh and Nielsen [2008]). Therefore, it may suggest that a simple change in cultivation condition and single genetic modification may not be enough to completely reroute the carbon flux from citric acid production to other organic acids. A series of genetic modifications may be required in the future work on metabolic engineering of *A. carbonarius* for production of valuable organic acids.

## Competing interests

The authors declare that they have no competing interests.

## Authors’ contributions

LY has made substantial contributions to conception and design, or acquisition of data, or analysis and interpretation of data; ML has been involved in discussing the results conducted and drafting the manuscript and revising it critically for important intellectual content; PL has been involved in all matters of the project (discussing the design of the experiments, the results and revision of the paper) and has given final approval of the version to be published. Each author has participated sufficiently in the work to take public responsibility for appropriate portions of the content. All authors read and approved the final manuscript.
